# Case Report: MRI, Clinical, and Pathological Correlates of Bromethalin Toxicosis in Three Dogs

**DOI:** 10.3389/fvets.2022.879007

**Published:** 2022-04-26

**Authors:** Vishal D. Murthy, Ehren McLarty, Kevin D. Woolard, Rell L. Parker, Gregg Kortz, Jamie N. King, Robert H. Poppenga, Marguerite F. Knipe, Peter J. Dickinson

**Affiliations:** ^1^Department of Veterinary Clinical Sciences, College of Veterinary Medicine, Washington State University, Pullman, WA, United States; ^2^Department of Surgical and Radiological Sciences, University of California, Davis, Davis, CA, United States; ^3^Department of Pathology, Microbiology and Immunology, University of California, Davis, Davis, CA, United States; ^4^Department of Small Animal Clinical Sciences, Virginia-Maryland Regional College of Veterinary Medicine, Virginia Polytechnic Institute and State University, Blacksburg, VA, United States; ^5^Department of Neurology, VCA Sacramento Veterinary Referral Center, Sacramento, CA, United States; ^6^California Animal Health and Food Safety Laboratory System, University of California, Davis, Davis, CA, United States

**Keywords:** biopsy, bromethalin, canine, corticospinal tract, desmethylbromethalin, leukoencephalopathy, restricted diffusion

## Abstract

Bromethalin toxicosis is an increasingly common clinical presentation in dogs that may be fatal depending on the extent of intoxication. Antemortem diagnosis of bromethalin toxicosis was achieved in three dogs by demonstration of the active metabolite desmethylbromethalin in fat or serum. Magnetic resonance imaging (MRI) findings were consistent with a diffuse leukoencephalopathy with restricted diffusion and prominent involvement of the corticospinal motor tracts on T2-weighted and diffusion-weighted sequences. Imaging findings were confirmed in one non-surviving dog at necropsy. Resolution of MRI abnormalities was demonstrated in one surviving dog that was consistent with the associated resolution of clinical signs. Initial findings in these dogs support further investigation of specific MRI patterns in cases of leukoencephalopathy to aid differential diagnosis. While antemortem detection of bromethalin and its metabolites confirms exposure, quantitation may be informative as a prognostic biomarker.

## Introduction

Rodenticide intoxications are one of the most common canine toxicoses, and the use of the over-the-counter rodenticide bromethalin has increased substantially the following action by the Environmental Protection Agency to phase out second-generation anticoagulant rodenticides (1–4). Bromethalin (N-methyl-2,4-dinitro-N-[2,4,6-tribromophenyl]-6-[trifluoromethyl] benzenamine) is a lipophilic diarylamine and weak acid that can locate within the mitochondrial inner membrane and act as a protonophore (5, 6). Bromethalin is a pro-pesticide that is converted by hepatic N-demethylation to the more active diphenylamine desmethylbromethalin, a potent central nervous system (CNS) toxicant with no antidote (7). Mitochondrial adenosine triphosphate (ATP) generation occurs through oxidative phosphorylation, comprised of two coupled processes: electron transport, generating a proton gradient across the inner mitochondrial membrane, and passage of protons through the ATP-synthase complex to create ATP (6). Desmethylbromethalin, a protonophore, uncouples these two processes by shuttling protons back across the inner membrane and discharging the electrochemical gradient, impairing ATP generation (6). CNS signs are commonly seen in both experimental and clinical cases (8–15). Pathology is consistent with diffuse spongy degeneration of predominantly CNS white matter with intramyelinic edema (7, 9–12, 16, 17), however, fatal intoxication with CNS signs and minimal histological lesions has been reported (18). Increased total brain water and sodium concentrations and intracranial hypertension have been documented experimentally in rats and dogs, and failure of ATP-dependent Na^+^-K ^+^ ion channel pumps has been proposed as a primary mechanism for cellular pathology (7, 15, 19). However, specific pathophysiology has not been defined, and other contributory mechanisms have also been proposed, such as increased lipid peroxidation and disruption of the blood-brain barrier (19).

Clinical signs of bromethalin intoxication are primarily neurological, however, gastrointestinal signs are also reported (7, 8, 13, 15, 20). In dogs, a “convulsive syndrome” (tremors, seizures, obtundation, and death) is reported at doses above the median lethal dose (LD50), while lower doses may result in a delayed “paralytic syndrome” characterized by muscle tremors, ataxia, paresis, and obtundation (15). Once severe signs of toxicosis, such as seizures, stupor, or coma, are seen, the prognosis is poor (15). Specific signs and severity may depend on several variables, such as species, total dose, drug absorption and metabolism, exposure time, and time to presentation. Cats appear to less commonly develop seizures as compared to dogs, and individual variability has been documented in experimental dogs receiving the same oral dose (15, 21). The LD50 of bromethalin varies by species within the range of 1–15 mg/kg (7, 13, 15). Cats and rats are more sensitive than dogs or rabbits, while guinea pigs are resistant to bromethalin (though not desmethylbromethalin) due to their inability to metabolize bromethalin to its more toxic metabolite (7). Clinical diagnosis of bromethalin intoxication is often presumptive based on the history of exposure and evidence of bait in feces or stomach contents (8). Magnetic resonance imaging (MRI) reports are limited, and a diffuse leukoencephalopathy with restricted diffusion based on diffusion-weighted imaging (DWI) has been reported in two cats (12). Definitive diagnosis, although infrequently utilized antemortem in the clinical setting (12, 14, 16, 18, 22), is through qualitative demonstration of desmethylbromethalin in tissues, such as adipose, kidney, liver, brain, or serum (23, 24), in conjunction with compatible clinical signs. The prognostic value of quantitative assessment of bromethalin and its metabolites in tissues and serum has not been determined in clinical cases.

In this case series, we report the antemortem assessment of desmethylbromethalin assays in three dogs with variable clinical outcomes and document MRI findings with marked similarities to those recently reported in cats with bromethalin intoxication (12). Specific MRI features and potential prognostic value of quantitative bromethalin assays are discussed in the context of clinical assessment of dogs exposed to bromethalin-based rodenticides.

### Case 1

A 10-year-old female spayed, 21 kg Catahoula Leopard Dog was presented for non-localized pain, following a 1-month progressive history of lethargy, inappetence, refusal to jump, and intermittent pacing and vocalization. Complete blood count (CBC), serum biochemistry, prothrombin time (PT), and partial thromboplastin time (PTT) were normal. Screening for heartworm disease, Lyme disease, ehrlichiosis, and anaplasmosis was negative. Thoracic and abdominal radiographs revealed no abnormalities. No history of trauma or toxicant exposure was reported.

At presentation, the dog was markedly reactive and aggressive on handling with non-localizable apparent pain and mild obtundation, interpreted as a manifestation of pain. The dog was ambulatory with no gait or postural abnormalities, and menace response was present bilaterally [oculus uterque (OU)]. Cranial nerve examination was unremarkable but spinal reflex testing was not possible. Definitive neuroanatomic localization was not possible, but lumbar pain was suspected. The abdominal ultrasound was unremarkable. MRI of the thoracolumbar vertebral column revealed multiple degenerative intervertebral discs and a region of contrast enhancement within the left sartorius muscle. Soft tissue injury or myositis could not be ruled out. A lumbar cerebrospinal fluid (CSF) sample was normal. While a definitive etiology was not identified, lumbar radicular pain was suspected based on the clinical presentation. Following anesthesia, the dog was observed to pace the kennel periphery and lean facing the wall. The patient was rested with trazodone (5 mg/kg q8 h) to facilitate confinement and prednisone (1 mg/kg q24 h) for nerve root pain. Clinical signs failed to resolve following 5 days of treatment and the owner reported episodes of getting stuck in corners. Cranial nerve examination and postural reactions were unremarkable at this time and a neuroanatomical localization of cerebral disease was made based on the head-pressing and behavior changes.

**Figure 1:**
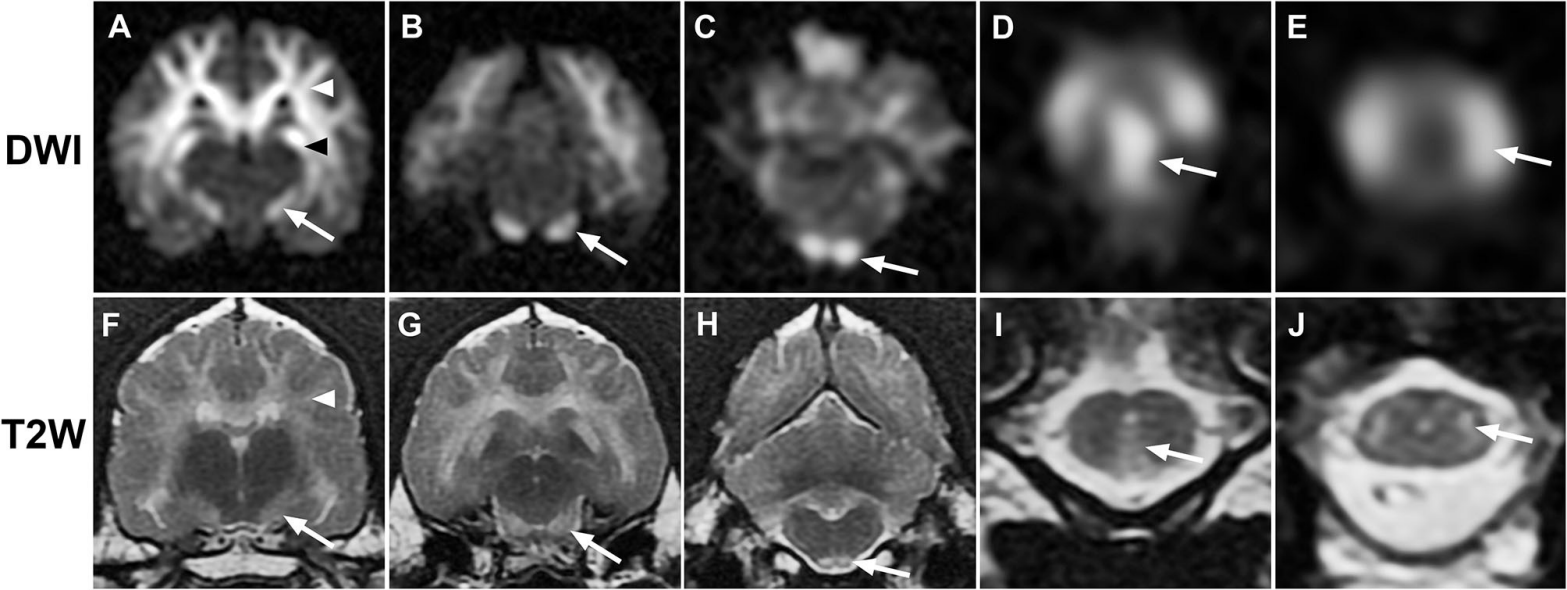
Sequential transverse MR images from case 1. **(A–E)**, diffusion-weighted (DWI); **(F–J)**, T2-weighted (T2W): Diffuse bilaterally symmetrical hyperintensity involving white matter tracts is present on DWI and T2W images. Hyperintensity consistent with the descending corticospinal tract (white arrows) is present at the level of the internal capsule **(A,F)**, midbrain crus **(B,G)**, pyramidal tracts **(C,H)**, pyramidal decussation **(D,I)**, and lateral corticospinal tracts **(E,J)**. Additional white matter tracts are defined, including corona radiata [**(A,F)**, white arrowheads] and hippocampal fornix [**(A)**, black arrowhead].

**Figure 2:**
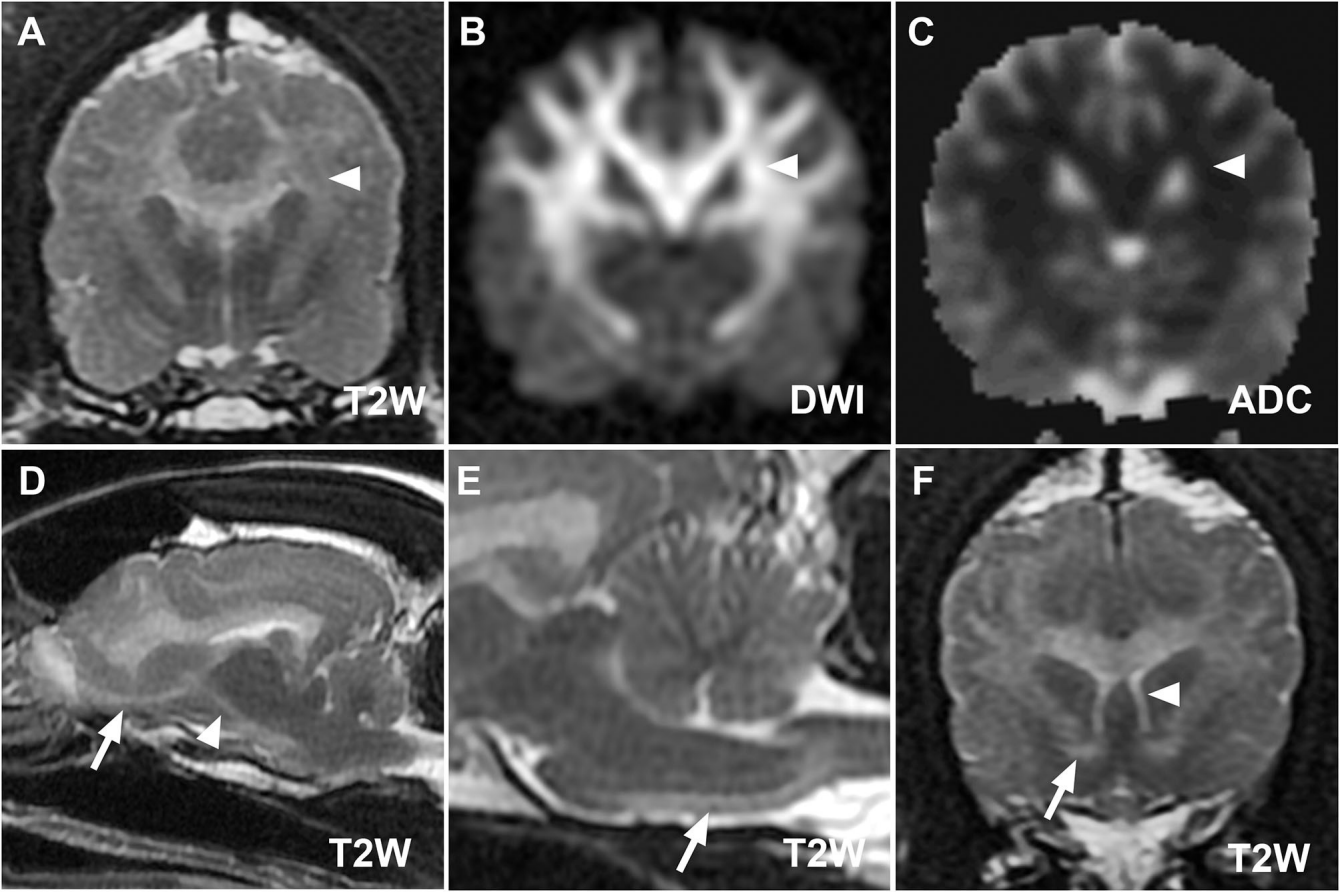
**(A–C)** MR images at the level of the thalamus from case 1 demonstrating restricted diffusion of defined lesions. Hyperintensity of corona radiata (white arrowheads) on T2W **(A)** and diffusion-weighted imaging (DWI) **(B)** images with hypointensity on the corresponding apparent diffusion coefficient map **(C)** is consistent with restricted diffusion. Parasagittal **(D)** and midsagittal T2W images **(E)** highlight the descending [**(D)**, white arrowhead] and pyramidal components [**(E)**, white arrow] of the corticospinal tract. The olfactory tract [**(D)**, white arrow], rostral commissure [**(F)**, white arrow, transverse T2W image], and columns of the fornix [**(F)**, white arrowhead] ventral to the hyperintense corpus callosum are also clearly defined.

MRI of the brain revealed extensive hyperintensity of cerebral and brainstem white matter on T2-weighted (T2W) and T2W fluid attenuated inversion recovery (FLAIR) sequences, with no contrast enhancement (Figures 1, 2). Lesions were prominent in the corona radiata, internal capsule, corpus callosum, fornix, olfactory tracts, and cerebellar peduncles. Elements of the corticospinal tracts (CST) were particularly prominent at the level of the brainstem and cranial cervical spinal cord. T2W hyperintense white matter structures also appeared hyperintense on diffusion-weighted imaging (DWI) (Figures 1, 2) and hypointense on the apparent diffusion coefficient (ADC) map (Figure 2), consistent with restricted diffusion. These findings suggested a diffuse leukoencephalopathy, such as bromethalin toxicosis. Transtentorial herniation and rostral cerebellar flattening were identified, consistent with intracranial hypertension. Under anesthesia, mean systemic systolic blood pressure by direct measurement was consistently below 120 mmHg (ref 90–160 mmHg). An ~10 g sample of dorsal subcutaneous lumbar fat was biopsied and submitted for desmethylbromethalin testing (23) at the California Animal Health and Food Safety Laboratory (CAHFS).

The dog recovered from general anesthesia and was started on phenobarbital (4 mg/kg PO q12 h) to reduce the risk of epileptic seizures. Prednisone (1 mg/kg/day) to treat the cerebral inflammation and trazodone (5 mg/kg q8 h) for confinement were continued. Over 5 days, the dog became less reactive to handling and touch, remained mildly obtunded, and was discharged on the above medications with instructions to taper and discontinue the corticosteroids over 1 week. In 2 weeks, the dog had returned to normal. Following normal neurological examination, trazodone was discontinued, and the phenobarbital was gradually tapered over 2 months and discontinued. Desmethylbromethalin testing was positive with a semi-quantitative concentration estimate of <0.1 ng/g based on comparison with a standard curve.

### Case 2

A 1.5-year-old intact female, 26 kg Labrador Retriever dog developed an acute onset of lethargy, progressive obtundation, hyper-reactivity to sound and touch, vocalization, hypersalivation, loss of urinary continence, and apparent trismus. No history of trauma or toxicant exposure was reported. CBC and serum biochemical profiles were normal. On presentation, general physical examination revealed fleas, otitis externa, and increased abdominal respiratory effort. On neurological examination, the dog was non-ambulatory tetraparetic with waxing and waning obtundation. Menace response was reduced OU with normal palpebral but absent corneal reflexes OU. Nasocortical response was reduced bilaterally. Masticatory muscle mass was normal. Mild anisocoria was noted with miosis of the left eye [oculus sinister (OS)] and intact direct and consensual pupillary light reflexes. Rotary nystagmus was induced on placing the dog in lateral recumbency, with a vertical, upbeat nystagmus in dorsal recumbency. A gag reflex could not be elicited. Postural reactions were absent in all limbs. Thoracic limb muscle tone was increased with normal myotatic reflexes and reduced withdrawal reflexes. Pelvic limb tone appeared normal with bilaterally hyper-reflexive patellar reflexes. A neuroanatomical localization of multifocal brain disease, characterized by the brainstem and cerebral disease, was made, with high concern for diffuse intracranial hypertension. Over the course of the examination, the dog developed opisthotonos.

**Figure 3:**
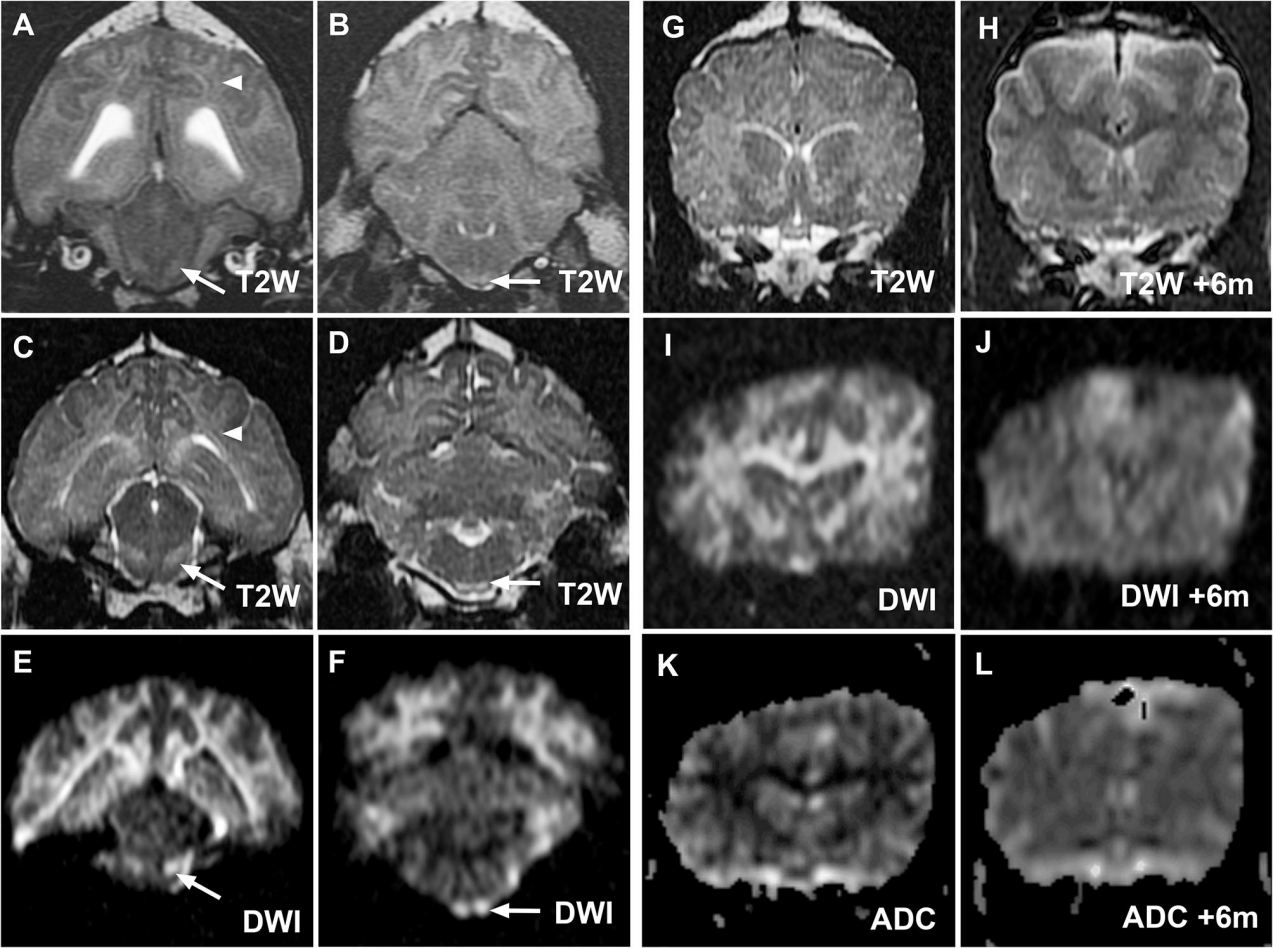
MR images from cases 2 **(A,B)** and 3 **(C–L)** demonstrating similar hallmark corticospinal tract lesions in both cases and demonstrating resolution of MRI changes in case 3. Transverse T2W images at the level of the midbrain and medulla oblongata for cases 2 **(A,B)** and 3 **(C,D)** demonstrate hyperintensity of corona radiata (white arrowheads) and prominence of the corticospinal tracts (white arrows) within the brainstem as in case 1. Lesions are again most noticeable on diffusion-weighted imaging (DWI) images from case 3 [**(E,F)**, white arrows]. Case 3 initial transverse T2W **(G)**, DWI **(I)** and apparent diffusion coefficient (ADC) map **(K)** images at the level of the caudate nuclei confirm restricted diffusion within white matter tracts as in case 1. Reversal of imaging abnormalities on repeated T2W **(H)**, DWI **(J)**, and ADC map **(L)** 6 months later was consistent with resolution of clinical signs.

MRI of the brain and cervical spinal cord (Figure 3) revealed generalized T2W and T2W-FLAIR hyperintensity of white matter tracts. Lesions were most prominent in the corona radiata, internal capsule, CST, and C1 white matter tracts. Diffuse hyperintensity was seen throughout the rest of the cervical spinal cord. Transtentorial and transforaminal cerebellar herniation were present. Faint meningeal and multifocal intra-axial cerebral contrast enhancement were noted. Findings were consistent with a diffuse leukoencephalopathy, such as bromethalin toxicosis with intracranial hypertension and syringohydromyelia. Under anesthesia, mean systemic blood pressure by direct measurement was consistently below 110 mmHg (ref 90–160 mmHg). The dog was treated with mannitol (0.5 g/kg IV) with concurrent intravenous (IV) fluid therapy and dexamethasone sodium phosphate (0.2 mg/kg IV). CSF collection was not attempted. An ~10 g sample of dorsal subcutaneous lumbar fat was biopsied and submitted for desmethylbromethalin testing at the CAHFS and the dog was recovered from anesthesia.

Following initial transient improvement in gait and mentation, a progressive decline was seen over 24 h with episodes of head pressing, vocalization, generalized hypertonia, opisthotonos, and loss of physiological nystagmus. The dog was treated with two additional doses of mannitol (0.5 g/kg IV), dexamethasone sodium phosphate (0.2 mg/kg IV), 2.5 mL/kg IV 7.2% hypertonic saline, and IV lipid emulsion therapy (15 mL/kg/h, reduced to 7.5 mL/kg/h following lipemia) (14, 22). The dog continued to decline and experienced respiratory arrest. Post-mortem lumbar CSF sampling revealed clear CSF with a mononuclear pleocytosis (127 cells/uL; ref <5 cells/uL), few erythrocytes (399/uL; ref 0/uL), and increased protein (37 mg/dL; ref <35 mg/dL). Desmethylbromethalin testing was positive with a semi-quantitative concentration estimate of >280 ng/g based on comparison to a standard curve. Necropsy revealed cerebellar herniation and diffusely turgid, flat cerebral gyri separated by shallow sulci. Histopathology revealed diffuse intramyelinic edema with segmental axonal swelling with gliosis. There was moderate syringohydromyelia, and dorsomedial and ventromedial white matter edema throughout the cervical and thoracic spinal cord. Additional anticoagulant rodenticide testing on the liver detected trace amounts of brodifacoum and increased levels of diphacinone (190 ppb), which were considered incidental findings in this dog, given the lack of hemorrhagic lesions.

### Case 3

A 5-year-old female spayed, 18 kg German Shepherd-cross dog developed acute behavior changes, panting, and ataxia. CBC, serum biochemistry panel, urinalysis, thoracic, and abdominal radiographs were unremarkable. Toxin exposure was considered possible. Activated charcoal with sorbitol (43 mL), IV lipid emulsion therapy (288 mL total), and 24 h of IV fluids were administered, transient improvement was seen, and the dog was discharged. Upon subsequent worsening of clinical signs, the dog was re-presented 4 days after the initial onset of clinical signs. The dog was obtunded with intermittent dysphoria, moderate generalized ataxia, circling to the left, and low head carriage. Other than an inconsistent menace response of the right eye (oculus dextrus: OD) and postural reaction deficits in all limbs, the neurological examination was normal. Due to concern for diffuse, potentially left-sided thalamocortical disease, MRI of the brain was performed (Figure 3) and revealed diffuse, bilaterally symmetrical T2W, T2W-FLAIR hyperintensity of the cerebral white matter tracts with no contrast enhancement. The white matter appeared hyperintense on DWI and hypointense on ADC maps (Figure 3). Abnormalities were most prominent in the CST, corona radiata, internal capsule, and corpus callosum. These findings suggested diffuse leukoencephalopathy, such as bromethalin toxicosis. Slight flattening of the caudal cerebellum was noted, with no signs of herniation. CBC, PT, and PTT were normal. Cisternal CSF analysis showed 4 nucleated cells/uL and total protein content of 30.4 mg/dL. Polymerase chain reaction (PCR) testing (Neurologic REALPCR™ PANEL, IDEXX, Westbrook, ME) of CSF for rickettsial, viral, fungal, and protozoal pathogens was negative. A serum sample was submitted to CAHFS for desmethylbromethalin testing.

The dog was treated with dexamethasone sodium phosphate (0.1 mg/kg IV), maropitant (1 mg/kg IV), and hospitalized overnight on IV fluid therapy. Mentation was improved but an inconsistent menace response OD, postural reaction deficits in all limbs, and intermittent circling to the left persisted. Desmethylbromethalin testing was positive with a semi-quantitative estimated concentration between 25 and 100 ng/g based on comparison with a standard curve. Following discharge on prednisone (0.5 mg/kg PO daily) for cerebral inflammation, maropitant (2 mg/kg PO daily) as an antiemetic and omeprazole (20 mg total PO q12 h) to reduce the risk of gastrointestinal ulceration, the dog returned to normal within 18 days of the initial onset of signs. Prednisone dose was tapered over 2 weeks, and the dog was neurologically normal with normal MRI 6 months later (Figure 3).

## Discussion

Three dogs were presented with multifocal neurological deficits and MRI consistent with a symmetrical, generalized leukoencephalopathy characterized by restricted diffusion and prominent involvement of the CST. Qualitative and semiquantitative evaluation of antemortem serum/fat desmethylbromethalin levels provided a diagnosis of bromethalin intoxication, and serum/tissue concentrations were broadly predictive of outcome consistent with experimental data. MRI findings in dogs appear to be similar to those previously reported in cats (12).

While a “neuron-centric” approach to CNS energy imbalance and excitotoxicity is often taken, oligodendroglia are highly sensitive, resulting in leukocentric disease presentations (25–28). A variety of human genetic and dog breed-related leukodystrophies have been reported, such as adult-onset human disorders (29–47); however, more relevant, non-breed related differentials in this case series included other toxicants known to disrupt oxidative phosphorylation (hexachlorophene, carbon monoxide (CO), and triethyltin) (6, 48–54) and systemic hypertension (55–58). Human hypertensive encephalopathy is associated with the leukotrophic syndrome posterior reversible encephalopathy (PRES), which is linked to other factors, such as eclampsia, chemotherapy, immune-mediated disease, immune suppression, renal disease, sepsis, and transplantation (57, 58). Somewhat confusingly, human acute toxic leukoencephalopathy (ATL), which is often reversible and characterized by periventricular restricted diffusion, is also reported in association with chemotherapeutics, heroin, cocaine, opioids, immunosuppressants, acute hepatopathy, and uremia (59, 60).

Less likely differential diagnoses for leukoencephalopathy based on history, signalment, bloodwork, and lesion distribution included radiation-associated encephalopathy (61), cobalamin and copper deficiency (62), hypotensive periventricular leukoencephalopathy (PVL) (63, 64), age-associated periventricular lesions (”leukoaraiosis“) (65), and leukocentric presentations of infectious or immune-mediated diseases, such as distemper (66), parvovirus (67), and granulomatous meningoencephalomyelitis (68). Progressive multifocal leukoencephalopathy associated with John Cunningham (JC) virus, (69), diffuse leukoencephalopathy associated with COVID-19 (70), and acute leukoencephalopathy with restricted diffusion associated with bacterial/viral infections are reported in humans (71), but not dogs. Hypoglycemia can predominantly affect white matter in humans (72), although gray matter involvement is common and such lesions may also predominate in CO exposure depending on exposure timing (49, 73, 74). Fumonisin B1 toxin (Fusarium spp.) is associated with leukoencephalomalacia in horses, but not documented in dogs (75, 76).

Imaging and pathology can be variable for many leukoencephalopathies and involvement of more restricted white matter regions and variable components of gray matter can be seen. Bromethalin-related pathology appears to be almost exclusively white matter-oriented. Restricted diffusion and prominence of CST were notable MRI findings in the described dogs and have additional diagnostic value in this context. Leukoencephalopathy with restricted diffusion, potentially reflecting cytotoxic pathology, is reported with toxic and metabolic causes, such as CO, organotin compounds, ATL, hypoglycemia, and PVL (49, 59, 60, 72, 77). Hypertensive encephalopathy and PRES are not generally associated with restricted diffusion, consistent with a vasogenic origin of MRI signal changes (55, 57, 58). Pronounced CST involvement, often with restricted diffusion, has been reported in humans with amyotrophic lateral sclerosis (ALS) (78, 79), cerebral insults (80, 81), chemotherapy (82), and a variety of human neonatal and adult syndromes of inborn errors of metabolism (29, 83). Conspicuous CST involvement is reported in some cases of Krabbe disease (84, 85), adrenoleukodystrophy (ALD) (86), and some mitochondriopathies (83, 87, 88); selective involvement of CST was reported in ~25% of mitochondrial leukodystrophy patients with brainstem involvement in one human study (87). Anatomical factors, including tract relative volume and length, may contribute to imaging findings; however, given the pathogenesis of bromethalin intoxication, the potential mitochondrial associations are intriguing. Mitochondrial defects have emerged as a common finding in ALS (78) and ALD-associated very-long-chain fatty acids have been shown to impair mitochondrial oxidative phosphorylation (89). Similarly, the accumulation of psychosine in Krabbe disease has been shown to interfere with mitochondrial electron transfer by altering the lipid membrane (90). Mechanisms for potentially increased sensitivity of CST neurons specifically are not defined, however, mitochondria are neither homogenous nor respond stereotypically in the same disease setting. ”Striking“ differences in mitochondrial replication, mitochondrial DNA copy number, and gene expression exist in different tissues (91), and recent data have shown that within the CNS, regulation of even basic mitochondrial functions differs between specific cell types and even neuronal subtypes providing potential mechanisms for ”selective vulnerability of specific neuronal populations“ during disease (92).

The LD50 for technical-grade (7) and rodenticide-based bromethalin (15) in dogs is reported at 4.7 and 3.65 mg/kg, respectively, with the lowest reported lethal dose of 2.5 mg/kg (15). American Society for the Prevention of Cruelty to Animals Animal Poison Control Center unpublished the data that documented deaths following bromethalin doses as low as 0.95 mg/kg [referenced in (93)]. Limited experimental data showed adipose levels of desmethylbromethalin of 49–325 ng/g following a lethal 6.25 mg/kg dose of bromethalin. Antemortem testing for bromethalin exposure is uncommon (8) but was essential in these cases where the specific history of exposure was lacking. Conclusions related to serum or fat biopsy concentrations and outcome are limited due to variable sources and timings of diagnostic samples, treatment regimens, and limited quantitative data available from the testing methodology (18, 23). However, reviewing available data from these and previously published experimental and clinical cases (Table 1), a predictable trend is apparent with sample desmethylbromethalin levels below those previously associated with bromethalin LD50 levels (9) being associated with a favorable outcome. No data relating serum levels to lethality and bromethalin dose in dogs are available, although a plasma elimination half-life of 5.6 days has been reported in mice (7).

**Table 1:** Reported desmethylbromethalin tissue levels and clinical outcomes in dogs.

	**Bromethalin dose mg/kg**	**Neurological signs**	**DMB level ng/g**	**Death**	**Reference**
3 Exp. Beagles	6.25	YES	FAT 49,58,325	YES	Dorman et al. (9)
Beagle	NA	Found dead^*^	FAT 390	YES	Romano et al. (18)
Case 1	NA	YES	FAT <0.1	NO	
Case 2	NA	YES	FAT >280	YES	
Norwich Terrier	NA	YES	SERUM 0.5	NO	Lyons et al. (14)
Pit Bull Terrier	0.17^**^	NO	SERUM 1–4^***^	NO	Heggem-Perry et al. (22)
Case 3	NA	YES	SERUM 25–100	NO	

* * normal within 24 h of death*.

** * owner reported*.

*** * Limit of detection*.

*Analysis in Dorman et al. (9) is quantitative; all other analyses are semiquantitative based on limited internal control samples*.

Neurological patients presented with MRI-defined diffuse leukoencephalopathy with restricted diffusion on DWI and ADC maps and prominent involvement of CST should have bromethalin intoxication as a major differential diagnosis in both dogs and cats. Serum or fat biopsies should be considered for both diagnosis and potential prognostic evaluation and given the lipophilic nature of desmethylbromethalin, an adipose tissue biopsy may provide the broadest diagnostic window (17). Prospective studies evaluating desmethylbromethalin in serum and adipose samples in a quantitative and temporal setting would be beneficial to further evaluate prognostic value.

## Data Availability Statement

The original contributions presented in the study are included in the article/supplementary material, further inquiries can be directed to the corresponding author.

## Author Contributions

VM and PD conceived of and wrote the manuscript. VM, MK, PD, RLP, GK, and JK provided cases. EM reviewed MRI. KW performed histopathology. RHP performed and advised on bromethalin assay. All authors have approved the final submitted version.

## Conflict of Interest

The authors declare that the research was conducted in the absence of any commercial or financial relationships that could be construed as a potential conflict of interest.

## Publisher's Note

All claims expressed in this article are solely those of the authors and do not necessarily represent those of their affiliated organizations, or those of the publisher, the editors and the reviewers. Any product that may be evaluated in this article, or claim that may be made by its manufacturer, is not guaranteed or endorsed by the publisher.
